# 5,6-Dimeth­yl-1,10-phenanthroline

**DOI:** 10.1107/S1600536813025087

**Published:** 2013-09-18

**Authors:** Sergio S. Rozenel

**Affiliations:** a Chemistry Department and Chemical Sciences Division of Lawrence Berkeley National Laboratory, University of California, Berkeley, California 94720, USA

## Abstract

In the title compound, C_14_H_12_N_2_, the N⋯N distance is 2.719 (1) Å. The N—C—C—N torsion angle [0.9 (1)°] is close to the ideal value of 0° as expected. Bond lengths and angles are consistent with those observed for [1,10]phenanthroline and coordinated 5,6 dimeth­yl[1,10]phenanthroline. In the crystal, C—H⋯N hydrogen bonds link the mol­ecules into *C*(4) chains running parallel to the *b* axis. Weak π–π inter­actions between benzene and pyridine rings [centroid–centroid distance = 3.5337 (7) Å] and between benzene rings [centroid–centroid distances = 3.6627 (7) and 3.8391 (7)Å] also occur.

## Related literature

For [1,10]phenanthroline and 5,6-dimeth­yl[1,10]phenan­thro­line, see: Ton & Bolte (2005 ▶) and Gasque *et al.* (1999 ▶), respectively. For hydrogen-bond motifs, see: Bernstein *et al.* (1995 ▶).
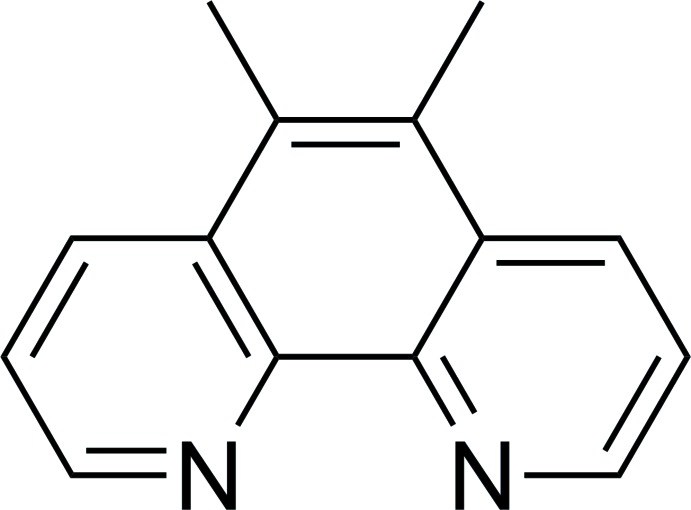


## Experimental

### 

#### Crystal data

C_14_H_12_N_2_
*M* *_r_* = 208.26
Monoclinic, 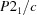

*a* = 7.1932 (7) Å
*b* = 10.0572 (10) Å
*c* = 13.8729 (13) Å
β = 93.673 (5)°
*V* = 1001.55 (17) Å^3^
*Z* = 4
Mo *K*α radiation
μ = 0.08 mm^−1^
*T* = 100 K
0.11 × 0.10 × 0.09 mm

#### Data collection

Bruker APEXII CCD diffractometer
Absorption correction: multi-scan (*SADABS*; Bruker, 2009 ▶) *T*_min_ = 0.991, *T*_max_ = 0.993
16500 measured reflections
1855 independent reflections
1613 reflections with *I* > 2σ(*I*)
*R*_int_ = 0.022

#### Refinement

*R*[*F* ^2^ > 2σ(*F* ^2^)] = 0.035
*wR*(*F* ^2^) = 0.100
*S* = 1.06
1855 reflections
171 parameters
H atoms treated by a mixture of independent and constrained refinement
Δρ_max_ = 0.16 e Å^−3^
Δρ_min_ = −0.20 e Å^−3^

### 

Data collection: *APEX2* (Bruker, 2009 ▶); cell refinement: *SAINT* (Bruker, 2009 ▶); data reduction: *SAINT*; program(s) used to solve structure: *SIR97* (Altomare, *et al.* 1999 ▶); program(s) used to refine structure: *SHELXL97* (Sheldrick, 2008 ▶); molecular graphics: *ORTEP-32* (Farrugia, 2012 ▶); software used to prepare material for publication: *WinGX* (Farrugia, 2012 ▶).

## Supplementary Material

Crystal structure: contains datablock(s) srd3013, I. DOI: 10.1107/S1600536813025087/bx2451sup1.cif

Structure factors: contains datablock(s) I. DOI: 10.1107/S1600536813025087/bx2451Isup2.hkl

Supplementary material file. DOI: 10.1107/S1600536813025087/bx2451Isup3.cml

Additional supplementary materials:  crystallographic information; 3D view; checkCIF report

## Figures and Tables

**Table 1:** Hydrogen-bond geometry (Å, °)

*D*—H⋯*A*	*D*—H	H⋯*A*	*D*⋯*A*	*D*—H⋯*A*
C2—H2⋯N1^i^	0.945 (14)	2.439 (13)	3.3718 (15)	169.0 (10)

Symmetry code: (i) 
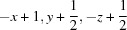
.

**Fig. 1.**
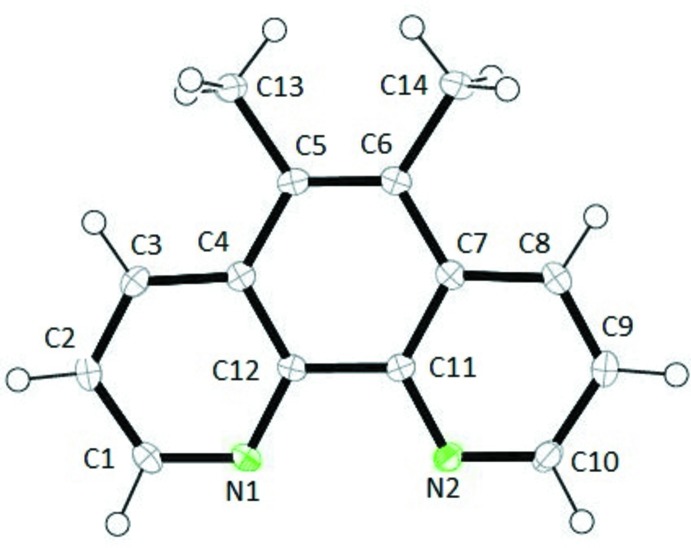
Top view of the structure of 5,6-dimethyl-1,10-phenanthroline, displacement ellipsoids drawn at the 50% probability level.

## supplementary crystallographic information

### 1. Comment

5,6-dimethyl-1,10-phenanthroline was obtained from Sigma-Aldrich. The distances and angles are consistent with those observed for [1,10]phenanthroline (Ton & Bolte, 2005) and coordinated 5,6-dimethyl-1,10-phenanthroline (Gasque, *et al.*, 1999). The crystal packing is stabilized by an intermolecular C—H···N hydrogen bond interaction which links the molecules into chains with graph-set notation *C*(4) (Bernstein, *et al.*, 1995) running parallel to the b axis. Weak intermolecular π-π interactions, involving the benzene rings, and the pyridine rings, respectively with an average plane-to plane separation of 3.5982 (7) Å further stabilize and reinforce the crystal structure.

### 2. Experimental

Crystals of the title compound, 5,6-dimethyl-1,10-phenanthroline, were obtained by sublimation at 160 °C under dynamic vacuum.

### 3. Refinement

All non-hydrogen atoms were refined anisotropically. Aromatic hydrogen atoms were located in the Fourier map and refined isotropically. Methyl hydrogen atoms were placed based on the expected geometry of the carbon atoms to which they were attached and refined using a riding model, with C—H distances 0.98 Å and U_iso_(H) = 1.5 U_eq_(C).

### Crystal data

**Table:**

C_14_H_12_N_2_	*F*(000) = 440
*M**_r_* = 208.26	SHELXL-97
Monoclinic, *P*2_1_/*c*	*D*_x_ = 1.381 Mg m^−^^3^
Hall symbol: -P 2ybc	Mo *K*α radiation, λ = 0.71073 Å
*a* = 7.1932 (7) Å	Cell parameters from 9819 reflections
*b* = 10.0572 (10) Å	θ = 2.5–25.4°
*c* = 13.8729 (13) Å	µ = 0.08 mm^−^^1^
β = 93.673 (5)°	*T* = 100 K
*V* = 1001.55 (17) Å^3^	Block, colourless
*Z* = 4	0.11 × 0.10 × 0.09 mm

### Data collection

**Table:**

Bruker APEXII CCD diffractometer	1855 independent reflections
Radiation source: microfocus sealed tube	1613 reflections with *I* > 2σ(*I*)
QUAZAR multilayer mirrors monochromator	*R*_int_ = 0.022
Detector resolution: 8.366 pixels mm^-1^	θ_max_ = 25.5°, θ_min_ = 2.5°
φ and ω scans	*h* = −8→8
Absorption correction: multi-scan (*SADABS*; Bruker, 2009)	*k* = −12→12
*T*_min_ = 0.991, *T*_max_ = 0.993	*l* = −16→16
16500 measured reflections	

### Refinement

**Table:**

Refinement on *F*^2^	Primary atom site location: structure-invariant direct methods
Least-squares matrix: full	Secondary atom site location: difference Fourier map
*R*[*F*^2^ > 2σ(*F*^2^)] = 0.035	Hydrogen site location: inferred from neighbouring sites
*wR*(*F*^2^) = 0.100	H atoms treated by a mixture of independent and constrained refinement
*S* = 1.06	*w* = 1/[σ^2^(*F*_o_^2^) + (0.0576*P*)^2^ + 0.2458*P*] where *P* = (*F*_o_^2^ + 2*F*_c_^2^)/3
1855 reflections	(Δ/σ)_max_ < 0.001
171 parameters	Δρ_max_ = 0.16 e Å^−^^3^
0 restraints	Δρ_min_ = −0.20 e Å^−^^3^

### Special details

**Table:**

Geometry. All e.s.d.'s (except the e.s.d. in the dihedral angle between two l.s. planes) are estimated using the full covariance matrix. The cell e.s.d.'s are taken into account individually in the estimation of e.s.d.'s in distances, angles and torsion angles; correlations between e.s.d.'s in cell parameters are only used when they are defined by crystal symmetry. An approximate (isotropic) treatment of cell e.s.d.'s is used for estimating e.s.d.'s involving l.s. planes.
Refinement. Refinement of *F*^2^ against ALL reflections. The weighted *R*-factor *wR* and goodness of fit *S* are based on *F*^2^, conventional *R*-factors *R* are based on *F*, with *F* set to zero for negative *F*^2^. The threshold expression of *F*^2^ > σ(*F*^2^) is used only for calculating *R*-factors(gt) *etc*. and is not relevant to the choice of reflections for refinement. *R*-factors based on *F*^2^ are statistically about twice as large as those based on *F*, and *R*- factors based on ALL data will be even larger.

### Fractional atomic coordinates and isotropic or equivalent isotropic displacement parameters (Å^2^)

**Table:**

	*x*	*y*	*z*	*U*_iso_*/*U*_eq_	
C1	0.42701 (15)	0.02490 (12)	0.23673 (8)	0.0163 (3)	
C2	0.42141 (15)	0.15376 (12)	0.19967 (8)	0.0169 (3)	
C3	0.35250 (15)	0.17126 (12)	0.10622 (8)	0.0155 (3)	
C4	0.29281 (14)	0.06112 (11)	0.04921 (8)	0.0133 (3)	
C5	0.22138 (14)	0.07469 (11)	−0.05047 (8)	0.0140 (3)	
C6	0.17602 (14)	−0.03576 (11)	−0.10406 (8)	0.0140 (3)	
C7	0.19526 (14)	−0.16667 (11)	−0.06034 (8)	0.0136 (3)	
C8	0.15222 (15)	−0.28403 (12)	−0.11278 (8)	0.0168 (3)	
C9	0.17425 (16)	−0.40516 (12)	−0.06876 (8)	0.0192 (3)	
C10	0.24086 (16)	−0.40937 (12)	0.02795 (9)	0.0193 (3)	
C11	0.26123 (14)	−0.18229 (11)	0.03706 (8)	0.0132 (3)	
C12	0.31061 (14)	−0.06488 (11)	0.09349 (8)	0.0128 (3)	
C13	0.20306 (16)	0.21432 (12)	−0.08965 (8)	0.0182 (3)	
H13A	0.3273	0.2519	−0.0961	0.027*	
H13B	0.1353	0.2692	−0.0452	0.027*	
H13C	0.1347	0.2125	−0.1530	0.027*	
C14	0.10570 (15)	−0.03036 (12)	−0.20896 (8)	0.0188 (3)	
H14A	−0.0264	−0.0549	−0.2148	0.028*	
H14B	0.1771	−0.0926	−0.2464	0.028*	
H14C	0.1207	0.0600	−0.2337	0.028*	
N1	0.37466 (12)	−0.08243 (9)	0.18696 (7)	0.0150 (2)	
N2	0.28440 (13)	−0.30292 (9)	0.08056 (7)	0.0163 (3)	
H1	0.4752 (17)	0.0073 (13)	0.3036 (9)	0.019 (3)*	
H2	0.4698 (17)	0.2240 (14)	0.2390 (9)	0.020 (3)*	
H3	0.3500 (18)	0.2604 (15)	0.0801 (9)	0.023 (3)*	
H8	0.1103 (18)	−0.2799 (14)	−0.1809 (10)	0.026 (3)*	
H9	0.1465 (17)	−0.4866 (14)	−0.1012 (9)	0.022 (3)*	
H10	0.2560 (18)	−0.4966 (14)	0.0606 (9)	0.024 (3)*	

### Atomic displacement parameters (Å^2^)

**Table:**

	*U*^11^	*U*^22^	*U*^33^	*U*^12^	*U*^13^	*U*^23^
C1	0.0132 (5)	0.0220 (7)	0.0137 (6)	−0.0005 (4)	0.0003 (4)	−0.0018 (5)
C2	0.0141 (6)	0.0184 (6)	0.0183 (6)	−0.0024 (4)	0.0026 (4)	−0.0061 (5)
C3	0.0146 (6)	0.0130 (6)	0.0194 (6)	0.0002 (4)	0.0043 (4)	0.0005 (5)
C4	0.0086 (5)	0.0157 (6)	0.0160 (6)	0.0004 (4)	0.0031 (4)	0.0004 (5)
C5	0.0095 (5)	0.0160 (6)	0.0167 (6)	0.0014 (4)	0.0030 (4)	0.0034 (5)
C6	0.0098 (5)	0.0184 (6)	0.0141 (6)	0.0011 (4)	0.0023 (4)	0.0007 (5)
C7	0.0093 (5)	0.0169 (6)	0.0149 (6)	0.0007 (4)	0.0020 (4)	−0.0005 (5)
C8	0.0154 (6)	0.0210 (7)	0.0142 (6)	−0.0015 (5)	0.0008 (4)	−0.0024 (5)
C9	0.0201 (6)	0.0157 (6)	0.0217 (6)	−0.0018 (5)	0.0014 (5)	−0.0045 (5)
C10	0.0209 (6)	0.0126 (6)	0.0241 (7)	0.0004 (5)	0.0001 (5)	0.0012 (5)
C11	0.0101 (5)	0.0145 (6)	0.0151 (6)	0.0005 (4)	0.0026 (4)	0.0012 (5)
C12	0.0091 (5)	0.0165 (6)	0.0130 (6)	0.0004 (4)	0.0019 (4)	0.0013 (4)
C13	0.0197 (6)	0.0169 (7)	0.0177 (6)	0.0004 (5)	0.0000 (5)	0.0029 (5)
C14	0.0192 (6)	0.0218 (7)	0.0150 (6)	0.0019 (5)	−0.0014 (4)	0.0023 (5)
N1	0.0138 (5)	0.0180 (5)	0.0131 (5)	0.0002 (4)	−0.0002 (4)	0.0002 (4)
N2	0.0172 (5)	0.0135 (5)	0.0178 (5)	−0.0001 (4)	−0.0003 (4)	0.0013 (4)

### Geometric parameters (Å, º)

**Table:**

C1—N1	1.3230 (15)	C8—C9	1.3673 (17)
C1—C2	1.3939 (17)	C8—H8	0.975 (14)
C1—H1	0.985 (13)	C9—C10	1.3960 (17)
C2—C3	1.3695 (16)	C9—H9	0.950 (14)
C2—H2	0.945 (14)	C10—N2	1.3220 (15)
C3—C4	1.4118 (16)	C10—H10	0.990 (14)
C3—H3	0.967 (15)	C11—N2	1.3605 (14)
C4—C12	1.4105 (16)	C11—C12	1.4483 (15)
C4—C5	1.4505 (16)	C12—N1	1.3595 (14)
C5—C6	1.3645 (16)	C13—H13A	0.9800
C5—C13	1.5085 (15)	C13—H13B	0.9800
C6—C7	1.4525 (16)	C13—H13C	0.9800
C6—C14	1.5108 (15)	C14—H14A	0.9800
C7—C8	1.4104 (15)	C14—H14B	0.9800
C7—C11	1.4118 (15)	C14—H14C	0.9800
			
N1—C1—C2	124.44 (10)	C8—C9—H9	122.8 (8)
N1—C1—H1	114.6 (8)	C10—C9—H9	118.6 (8)
C2—C1—H1	121.0 (7)	N2—C10—C9	124.10 (11)
C3—C2—C1	117.96 (10)	N2—C10—H10	116.7 (8)
C3—C2—H2	123.3 (8)	C9—C10—H10	119.2 (8)
C1—C2—H2	118.7 (8)	N2—C11—C7	123.22 (10)
C2—C3—C4	120.54 (11)	N2—C11—C12	117.93 (10)
C2—C3—H3	118.1 (8)	C7—C11—C12	118.84 (10)
C4—C3—H3	121.3 (8)	N1—C12—C4	123.21 (10)
C12—C4—C3	116.43 (10)	N1—C12—C11	117.78 (10)
C12—C4—C5	121.10 (10)	C4—C12—C11	119.00 (10)
C3—C4—C5	122.46 (10)	C5—C13—H13A	109.5
C6—C5—C4	120.03 (10)	C5—C13—H13B	109.5
C6—C5—C13	123.31 (10)	H13A—C13—H13B	109.5
C4—C5—C13	116.65 (10)	C5—C13—H13C	109.5
C5—C6—C7	119.83 (10)	H13A—C13—H13C	109.5
C5—C6—C14	123.31 (10)	H13B—C13—H13C	109.5
C7—C6—C14	116.87 (10)	C6—C14—H14A	109.5
C8—C7—C11	116.73 (10)	C6—C14—H14B	109.5
C8—C7—C6	122.09 (10)	H14A—C14—H14B	109.5
C11—C7—C6	121.17 (10)	C6—C14—H14C	109.5
C9—C8—C7	119.99 (10)	H14A—C14—H14C	109.5
C9—C8—H8	119.4 (8)	H14B—C14—H14C	109.5
C7—C8—H8	120.6 (8)	C1—N1—C12	117.36 (9)
C8—C9—C10	118.62 (11)	C10—N2—C11	117.34 (10)

### Hydrogen-bond geometry (Å, º)

**Table:**

*D*—H···*A*	*D*—H	H···*A*	*D*···*A*	*D*—H···*A*
C2—H2···N1^i^	0.945 (14)	2.439 (13)	3.3718 (15)	169.0 (10)

Symmetry code: (i) −*x*+1, *y*+1/2, −*z*+1/2.
